# Relationship between methylenetetrahydrofolate reductase C677T gene polymorphism and neutrophil gelatinase-associated lipocalin in early renal injury in H-type hypertension

**DOI:** 10.1186/s12872-024-03704-6

**Published:** 2024-01-18

**Authors:** Chi Zhang, Qiu-Ping Xin, Yun-BO Xie, Xiang-Yu Guo, En-Hong Xing, Zhi-Jie Dou, Cui Zhao

**Affiliations:** 1 Department of Neurology, Affiliated Hospital of Chengde Medical University, No. 36 of Nanyingzi Street, Shidongzigou District, Chengde, 067000 China; 2 Department of General Practice, Affiliated Hospital of Chengde Medical University, No. 36 of Nanyingzi Street, Shidongzigou District, Chengde, 067000 China; 3 Central Laboratory, Affiliated Hospital of Chengde Medical University, Chengde, 067000 China

**Keywords:** H-type hypertension, MTHFR C677T gene, Polymorphism, Early kidney injury, NGAL

## Abstract

**Objective:**

To analyse the relationship between the polymorphisms of the H-type hypertensive methylenetetrahydrofolate reductase (MTHFR) C677T gene and neutrophil gelatinase-associated lipocalin (NGAL) in early kidney injury.

**Method:**

A total of 279 hospitalised patients with hypertension were selected and grouped according to their homocysteine (Hcy) level. If their blood Hcy level was ≥ 10 µmol/L they were assigned to the H-type hypertensive group, and if it was < 10 µmol/L they were assigned to the non-H-type hypertensive group. Blood lipid indexes, renal function indexes and blood glucose indexes were collected, and the differences between the two groups were compared. Furthermore, MTHFR C677T genotype distribution and allele frequency and Hcy level of MTHFR C677T genotype were compared, and logistic multiple regression analysis was conducted for the correlation of different genotypes of MTHFR C677T and the early kidney injury marker NGAL.

**Results:**

In the non-H-type hypertensive group, the levels of Hcy and NGAL, cystatin, blood urea nitrogen, serum creatinine, uric acid, serum β2-microglobulin and urinary microalbumin-to-creatinine ratio increased significantly, and the glomerular filtration rate level decreased significantly, when compared with the H-type hypertensive group, with statistical differences (*p* < 0.05). The H-type hypertensive group and the non-H-type hypertensive group had significant differences in the CC, CT and TT genotypes and allele frequencies at the MTHFR C677T locus. The MTHFR C677T gene mutation rate of the H-type hypertensive group was significantly higher than that of the non-H-type hypertensive group. The H-type hypertensive group had higher levels of the TT genotype and CT genotype Hcy. There was a statistical difference (*p* < 0.05).

**Conclusion:**

Methylenetetrahydrofolate reductase C677T polymorphism is correlated with the Hcy level, and its gene polymorphism will affect the Hcy level. Methylenetetrahydrofolate reductase C677T polymorphism has an interactive effect with NGAL. Screening NGAL and reducing Hcy levels are valuable methods for the prevention and treatment of early renal injury in patients with H-type hypertension and help improve the prognosis of patients and their quality of life.

## Introduction

More than 75% of patients with hypertension in China have increased levels of homocysteine (Hcy), which is considered H-type hypertension when Hcy ≥ 10 µmol/L [1]. Patients with H-type hypertension are more susceptible to osteoporosis [2] and to stroke [3]. Methylenetetrahydrofolate reductase (MTHFR) plays a critical role in Hcy metabolism [4]. Homocysteine plays a pivotal role in various biochemical processes, including DNA methylation, nucleotide synthesis and protein function. Methylenetetrahydrofolate reductase catalyses the conversion of 5,10-methylenetetrahydrofolate to 5-methyltetrahydrofolate, a biologically active form of folate. The most common mutation in the MTHFR gene is the C677T polymorphism, which results in the substitution of alanine for valine at position 222 of the enzyme. This genetic variation has been associated with reduced MTHFR enzyme activity, resulting in the accumulation of Hcy in the bloodstream [5]. Elevated Hcy levels, particularly in individuals with the TT genotype, have been associated with an increased risk of hypertension [6]. Furthermore, the aberrant accumulation of Hcy can lead to kidney damage, affecting renal function [7]. The intricate interplay between the biochemical role of MTHFR and its genetic variations highlights the importance of understanding these mechanisms in the context of diseases such as H-type hypertension.

Recent studies have shown that neutrophil gelatinase-associated lipocalin (NGAL) is a sensitive biomarker of early renal structural damage and is closely related to Hcy levels in patients with H-type hypertension [8,9]. Many domestic and foreign studies involving the relationship between H-type hypertension and renal injury take β2-microglobulin (β2-MG), cystatin (Cys-C), urinary microalbumin-to-creatinine ratio (ACR) and glomerular filtration rate (GFR) as research indexes. This study investigates the relationship between H-type hypertension and early renal injury factor NGAL by taking the MTHFR C677T gene locus in patients with H-type hypertension as an entry point and analyses the association between NGAL and MTHFR C677T gene polymorphism to provide some reference for clinical early renal injury research, as well as providing new ideas for the prevention and treatment of target organ damage in H-type hypertension.

## Materials and methods

### General information about patients

A total of 278 patients with hypertension who were admitted to the general medical department of our hospital (to ensure a homogeneous and controlled cohort) between October 2020 and October 2021 were selected and grouped according to their Hcy levels. After power analysis, the number of patients met the requirements for carrying out the study. Patients with blood Hcy ≥ 10 µmol/L were considered patients with H-type hypertension and were assigned to the H-type hypertensive group; patients with blood Hcy < 10 µmol/L were considered control patients with hypertension and were assigned to the non-H-type hypertensive group. The inclusion criteria were as follows: The patients met the diagnostic criteria for hypertension as defined in the 2019 Chinese Guidelines for the Prevention and Treatment of Hypertension [10]; the patients and their families provided informed consent. The study was conducted in accordance with the Declaration of Helsinki and was approved by the hospital ethics committee (approval date: 12 October 2020, ethical batch number: LL2021094). The criteria for early renal impairment were as follows [11]: The patients were clinically asymptomatic; the patients had an ACR of 30–300 mg/g. The exclusion criteria were as follows: The patients had taken vitamin B12, vitamin B6, folic acid or other factors affecting serum Hcy levels in the past 3 months; the patients had combined thyroid disease or malignant tumour disease; the patients had secondary hypertension, such as renal parenchymal hypertension, primary aldosteronism or renal artery stenosis (Table 1). It is important to note that some of these cases were previously used in an earlier study by the authors [12]. Although there is an overlap in the selection of cases between these two papers, this research focuses on a different aspect. To avoid data duplication, the cases in the present study were reanalysed and independent results and discussions have been provided.

**Table 1:** Comparison of baseline data between the two groups ($\bar x \pm s$)

Clinical data	H-type hypertensive group (*n* = 137)	non-H-type hypertensive group (*n* = 142)	T/χ ^2^	*P*
Male (n, %)	96 (70.68)	101 (72.62)	0.083	0.772
Age (years)	66.90 ± 3.44	66.89 ± 3.24	0.024	0.981
BMI (kg/m ^2^)	24.54 ± 2.07	23.71 ± 2.46	0.613	0.540
Height (m)	1.6580 ± 0.0721	1.6604 ± 0.0745	0.269	0.782
Systolic blood pressure (mmHg)	166.10 ± 16.25	167.65 ± 15.82	0.792	0.427
Diastolic Blood Pressure (mmHg)	95.29 ± 10.49	95.03 ± 9.66	0.211	0.742
Heart rate (bmp)	82.00 ± 9.00	81.00 ± 8.00	0.081	0.711
Hemoglobin (g/L)	5.71 ± 1.44	5.67 ± 1.42	0.991	0.851
HbA1c (%)	13.61 ± 1.28	13.98 ± 1.31	1.729	0.099
TC (mmol/L)	4.03 ± 1.12	4.18 ± 1.43	0.958	0.832
TG (mmol/L)	1.85 ± 0.80	1.89 ± 0.72	0.432	0.666
HDL-C (mmol/L)	2.13 ± 0.36	2.11 ± 0.37	0.450	0.653
LDL-C (mmol/L)	2.34 ± 0.28	2.40 ± 0.31	1.667	0.097
GLU (mmol/L)	7.08 ± 0.12	7.06 ± 0.08	1.616	0.107

Note: BMI: body mass index; TG: triacylglycerol; TC: total cholesterol; LDL-C: Low-Density Lipoprotein Cholesterol; HDL-C: High-Density Lipoprotein Cholesterol; GLU: fasting blood glucose

### Blood sampling

Blood lipid, blood glucose and renal function parameters were collected and compared between the two groups; the MTHFR C677T genotype distribution, allele frequency and the Hcy levels of each genotype of MTHFR C677T were also compared; logistic multiple regression analysis was performed to analyse the correlation between the different genotype distributions of MTHFR C677T and NGAL, which is a marker of early renal injury.

### Biochemical indicators

Patients underwent serum collection in the morning on an empty stomach. Vacuum lancets were used to collect fresh blood into sterile tubes and were allowed to stand at room temperature for 1 h. The collected blood was centrifuged at 2,500 rpm for 10 min at 4℃, and the upper serum was collected. The collected serum was tested using an automatic biochemical analyser (Hitachi 7600, Tokyo, Japan). The test parameters included Cys-C, triacylglycerol, total cholesterol, blood urea nitrogen (BUN), fasting blood glucose, uric acid (UA), low-density lipoprotein cholesterol and serum creatinine (CRE). The formula used for GFR was as follows: men: (140 – age [years]) × body weight (kg) x 1.23 / CRE concentration (µmol/L); women: (140 – age [years]) × body weight (kg) x 1.03 / CRE concentration (µmol/L). The normal estimated GFR ranged from 90 to 120 mL/min/1.73 m^[2 [13]]^.

### Genomic deoxyribonucleic acid extraction

On the morning after the day of admission, 5 mL of cubital venous blood was drawn from the patients in the two groups under a fasting state, stored in EDTA anticoagulant blood collection tubes and cryopreserved at − 80 °C.

The detection of the C677T polymorphism of the MTHFR gene was performed as follows: The C677T polymorphism of the MTHFR gene was detected by the fluorescence quantitative method. The kit was provided by Tyler Medical Co., Ltd. (Tianjin, China), and genomic DNA was extracted and detected according to the kit instructions. Venous blood samples (50 µL) that were collected previously were removed, and genomic DNA was extracted from leukocytes by whole blood. The MTHFR C677T gene fragment was amplified by the amplification refractory mutation system polymerase chain reaction (ARMS-PCR).

### Amplification refractory mutation system polymerase chain reaction

Polymerase chain reaction conditions: unwinding at 94℃ for 10 min; denaturation at 94℃ for 45 s, annealing at 62℃ for 40 s and extension at 72℃ for 45 s, for a total of 39 cycles; extension at 72℃ for 7 min. The quantitative fluorescence detection used a Taqman™ MGB probe assay. The amplified products were digested with Hinf (10U) (Takara, Tokyo, Japan), and the digested products were separated by adding 2% agarose gel electrophoresis (the DNA fragment length was 200 bp after digestion) and then stained with ethidium bromide to observe and analyse the DNA chromogenic bands and determine the genotype. The sequences of the primers were as follows: F: GAGCGGCATGAGAGACTCC; R: CCGGTCAAACCTTGAGATGAG.

### Enzyme-linked immunosorbent assay

Detection of Hcy and β2-MG: Plasma Hcy and β2-MG levels were measured using an enzyme-linked immunosorbent assay (ELISA) kit (Beijing Jiuqiang Biotechnology Co. Ltd., Beijing, China) using a DG 022 A enzyme-linked immunosorbent assay instrument. The detection range of the ELISA kit for Hcy was 0.5–25 µmol/L; the lowest detection concentration was 0.1 µmol/L; repeatability: The intra-plate coefficient of variation was < 10% and the inter-plate coefficient of variation was < 15%; each experiment was repeated three times. The detection range of the ELISA kit for β2-MG was 12.5–400 ng/mL; the lowest detection concentration was 1.0 ng/mL; repeatability: The intra-plate coefficient of variation was < 10% and inter-plate coefficient of variation was < 15%; each experiment was repeated three times.

NGAL detection: The collected serum was centrifuged at 4,000 r/min for 10 min, and the supernatant was taken to detect the serum NGAL level using an ELISA kit (RampD, MA, USA), which was operated strictly according to the instructions; the results were averaged over the values of the duplicate wells. The detection range of the ELISA kit for NGAL was 3.75–120 ng/mL; the lowest detection concentration was 0.1 ng/mL; repeatability: The intra-plate coefficient of variation was < 10% and inter-plate coefficient of variation was < 15%; each experiment was repeated three times.

### Microalbumin to creatinine ratio assay

Mid-morning urine samples were collected from patients and tested by immunoturbidimetry using an ACR-300-Automatic Urine Microalbumin Creatinine Analyzer (Pumen, Shenzhen, China).

### Statistical analysis

The SPSS 26.0 software package was used for analysis. The normality of the data distribution was tested using histograms. All data had normality and homogeneity of variance and were expressed as mean ± standard deviation ($\bar x \pm s$); the chi-squared test or *t*-test was used for the comparison of data between groups; multivariate logistic regression was used for multivariate analysis. The chi-squared test was applied in the statistical inference of categorical data, including the comparison of two rates or two constituent ratios, the comparison of multiple rates or multiple constituent ratios and the correlation analysis of categorical data. The *t*-test was mainly used for normal distribution with a small sample size and unknown overall standard deviation. A *p*-value < 0.05 indicated a statistically significant difference. The online software SHEsis (http://analysis.bio-x.cn/myAnalysis.php) was used to test whether the genotype frequency distribution was in Hardy–Weinberg equilibrium (HWE) and whether *p* > 0.05. The analysis showed that the distribution was in HWE equilibrium, indicating that the sample population of this experiment was well-represented without selection bias.

## Results

### Comparison of general data of patients between the two groups

As shown in Table 1, there were no statistically significant differences in gender, age, systolic blood pressure, diastolic blood pressure, blood glucose or blood lipid parameters between the two groups (*p* > 0.05).

### Comparison of homocysteine and neutrophil gelatinase-associated lipocalin levels between the two groups

As shown in Table 2, compared with the non-H-type hypertensive group, the levels of Hcy and NGAL in the H-type hypertensive group were higher, and the difference was statistically significant (*p* < 0.05).

**Table 2:** Comparison of Hcy and NGAL levels between the two groups ($\bar x \pm s$)

	H-type hypertensive group (*n* = 137)	non-H-type hypertensive group (*n* = 142)	T/χ ^2^	*P*
Hcy (µmol/L)	20.61 ± 3.54	4.19 ± 2.75	16.371	< 0.001
NGAL (mmol/L)	60.33 ± 21.08	53.91 ± 17.43	2.731	0.006

Note: NGAL: Neutrophil gelatinase-associated lipocalin. Hcy: homocysteine

### Comparison of renal function indicators between the two groups

As shown in Table 3, the levels of Cys-C, BUN, CRE, UA, CRE, β2-MG and ACR were significantly increased, the GFR level was significantly lower in the H-type hypertensive group compared with the non-H-type hypertensive group, and the differences were statistically significant (*p* < 0.05).

**Table 3:** Comparison of renal function indicators between the two groups ($\bar x \pm s$)

Renal function indicators	H-type hypertensive group (*n* = 137)	non-H-type hypertensive group (*n* = 142)	T/χ ^2^	*P*
GFR mL/(min·1.73 m^2^)	82.16 ± 10.33	85.30 ± 8.43	2.741	0.006
Cys-C (mmol/L)	1.29 ± 0.55	0.79 ± 0.14	10.301	< 0.001
BUN (mmol/L)	7.43 ± 2.70	5.71 ± 1.12	6.832	< 0.001
CRE (mg/dl)	6.14 ± 1.86	2.54 ± 1.04	19.700	< 0.001
UA (µmol/L)	346.88 ± 115.62	307.30 ± 76.02	3.333	0.009
β2-MG (mg/L)	2.01 ± 0.58	1.34 ± 0.43	10.804	< 0.001
ACR (mg/g)	58.16 ± 5.36	49.60 ± 4.12	9.174	< 0.001

Note: GFR: Glomerular filtration rate; Cys-C: cystatin; BUN: Blood urea nitrogen; CRE: Serum creatinine; UA: Uric acid; β2-MG: β2-microglobulin; ACR: Creatinine ratio

### Comparison of methylenetetrahydrofolate reductase C677T genotype and allele frequencies between the two groups

As shown in Table 4, there were significant differences in the CC, CT and TT genotypes and allele frequencies at the MTHFR C677T locus between the H-type hypertensive group and the non-H-type hypertensive group (*p <* 0.05), and the MTHFR C677T gene polymorphism in the H-type hypertensive group was significantly higher than that in the non-H-type hypertensive group (*p <* 0.05).

**Table 4:** Comparison of genotype and allele frequencies of MTHFR C677T between the two groups (n,%)

	H-type hypertensive group (*n* = 137)	non-H-type hypertensive group (*n* = 142)	T/χ ^2^	*P*
Genotype Frequency				
CC	7 (5.11)	32 (22.54)	7.222	<0.001
CT	31 (22.63)	76 (53.52)		
TT	99 (72.26)	34 (23.94)		
Allele frequency				
C	45 (16.42)	140 (49.30)	11.439	<0.001
T	229 (83.58)	144 (50.70)		

### Comparison of homocysteine levels between the two groups of methylenetetrahydrofolate reductase C677T genotype

As shown in Table 5, the TT, CT and CC genotypes had higher Hcy levels in the H-type hypertensive group compared with those in the non-H-type hypertensive group, with statistically significant differences (*p* < 0.05).

**Table 5:** Comparison of Hcy (µmol/L) levels between the two groups of MTHFR C677T genotype ($\bar x \pm s$)

Genotype	H-type hypertensive group (*n* = 137)	non-H-type hypertensive group (*n* = 142)	T/χ ^2^	*P*
CC	17.36 ± 1.28	16.97 ± 1.48	2.313	0.021
CT	18.64 ± 3.08	14.15 ± 2.67	12.810	< 0.001
TT	20.37 ± 4.16	15.71 ± 3.81	9.603	< 0.001

### Multiple linear regression analysis of methylenetetrahydrofolate reductase C677T gene polymorphism and neutrophil gelatinase-associated lipocalin

As shown in Table 6, the TT genotype and CT genotype were strongly associated with NGAL compared with the CC genotype, and there was an interaction. The difference was statistically significant (*p* < 0.05).

**Table 6:** Multiple linear regression analysis of MTHFR C677T gene polymorphism and NGAL in the two groups ($\bar x \pm s$)

Variable	NGAL		
	β	SE	*P*
MTHFR C677T			
CC	1.00	-	-
CT	-0.065	0.015	< 0.001
TT	-0.042	0.020	< 0.001

Note: NGAL: Neutrophil gelatinase-associated lipocalin

## Discussion

Hypertension and hyperhomocysteinemia (i.e. H-type hypertension) usually occur simultaneously in the Chinese hypertensive population [14]. This study unveils a significant association between MTHFR C677T polymorphism, elevated Hcy levels and early renal injury in H-type hypertension. Patients with H-type hypertension exhibit higher frequencies of CC, CT and TT genotypes at the MTHFR C677T locus, accompanied by increased Hcy levels. Notably, early renal injury markers, including NGAL, are elevated in patients with H-type hypertension compared with patients with common hypertension. These findings highlight the intricate interplay of genetic factors, Hcy metabolism and renal damage in the context of H-type hypertension, providing valuable insights for future research and potential therapeutic interventions.

The metabolic pathways of Hcy involve various enzymes, with MTHFR C677T standing out as a crucial player in its metabolism, attracting considerable attention in clinical research [15–17]. Concurrently, studies have highlighted NGAL as a significant marker for diagnosing early kidney injury, playing a pivotal role in predicting mortality and gauging the severity of kidney disease [18,19]. The kidney, as a primary target organ of hypertensive damage, is significantly impacted by the abnormal elevation of Hcy. This elevation contributes to renal damage and a decline in renal function and exacerbates the elevated Hcy scenario in patients with H-type hypertension, creating a detrimental cycle [20–22]. The study’s results indicate substantial increases in Cys-C, β 2 -MG, ACR levels and Hcy levels in patients with H-type hypertension compared with those with common hypertension. This implies a higher susceptibility of patients with H-type hypertension to early renal injury, where Hcy levels serve as crucial evaluative markers.

Considering the distribution of the MTHFR gene, with its highest activity in the kidney, it can be inferred that during early renal injury in H-type hypertension, glomerular filtration and metabolic function are compromised, leading to increased Hcy levels. Being an active enzyme in Hcy metabolism, MTHFR likely plays a regulatory role in renal function [23,24]. The study further demonstrates significantly increased NGAL levels in patients with H-type hypertension compared with individuals with common hypertension.

The MTHFR C677T gene polymorphism is the most common genetic determinant of elevated Hcy levels, and CC, CT and TT genotypes are the three locus genes of MTHFR C677T. Our study unveils significant disparities in the TT, CT and CC genotypes and allele frequencies between patients with H-type hypertension and their counterparts with common hypertension. The heightened significance of the MTHFR C677T gene polymorphism in H-type hypertension is evident. In comparison with common hypertension patients, those with H-type hypertension exhibit elevated Hcy levels, specifically associated with the TT and CT genotypes. Furthermore, our multiple linear regression analysis probing the relationship between MTHFR C677T gene polymorphism and NGAL reveals a robust association of the TT and CT genotypes with NGAL, surpassing the CC genotype. This interaction underscores the correlation between MTHFR C677T polymorphism, Hcy levels and the early onset of kidney injury in H-type hypertension.

In light of these findings, screening for NGAL and proactive measures to reduce Hcy levels emerge as pivotal strategies for preventing and managing early renal injury in patients with H-type hypertension. While folic acid therapy is recognised for its role in influencing Hcy metabolism and may contribute to prevention, it is unlikely to be sufficient as a standalone solution [25]. The impact of the MTHFR C677T gene polymorphism on Hcy levels suggests the potential benefits of a more personalised approach, including gene therapy. This could address the underlying genetic factors contributing to elevated Hcy and hypertension. A comprehensive strategy should encompass lifestyle modifications, such as a balanced diet and regular exercise, along with blood pressure management. Recognising individual variability, treatment plans should be tailored based on genetic profiling and relevant clinical factors. Ongoing research is crucial to further understand the intricate mechanisms linking the MTHFR gene, Hcy metabolism and hypertension, refining preventive strategies.

While our study contributes valuable insights, certain limitations should be acknowledged. The relatively small sample size may affect the generalisability of our findings. Further studies with larger and more diverse cohorts are necessary to validate our results. Additionally, the cross-sectional design of the study limits our ability to establish causal relationships. Longitudinal studies are warranted to provide a more comprehensive understanding of the dynamic interplay between MTHFR C677T polymorphism, Hcy levels and early renal injury in H-type hypertension.

## Conclusion

In summary, there is an association between the MTHFR C677T polymorphism and Hcy levels, and mutations in its gene can affect Hcy levels. Methylenetetrahydrofolate reductase C677T polymorphism interacts with NGAL, and screening NGAL and reducing Hcy levels are important means to prevent and treat early renal injury in patients with H-type hypertension.

## Data Availability

The data generated and analysed in the presented study are available from the corresponding author on request.
